# Knowledge and information needs of young people with epilepsy and their parents: Mixed-method systematic review

**DOI:** 10.1186/1471-2431-10-103

**Published:** 2010-12-31

**Authors:** Sheila A Lewis, Jane Noyes, Stephen Mackereth

**Affiliations:** 1 Room 1021, 1st Floor, Glan Clwyd Hospital, Bodelwyddan LL18 5UJ, UK; 2 Centre for Health-Related Research, Bangor University, Bangor LL57 2EF, UK; 3 Royal Alexandra Hospital, Marine Drive, Rhyl, Denbighshire LL18 3AS, UK

## Abstract

**Background:**

Young people with neurological impairments such as epilepsy are known to receive less adequate services compared to young people with other long-term conditions. The time (age 13-19 years) around transition to adult services is particularly important in facilitating young people's self-care and ongoing management. There are epilepsy specific, biological and psycho-social factors that act as barriers and enablers to information exchange and nurturing of self-care practices. Review objectives were to identify what is known to be effective in delivering information to young people age 13-19 years with epilepsy and their parents, to describe their experiences of information exchange in healthcare contexts, and to identify factors influencing positive and negative healthcare communication.

**Methods:**

The Evidence for Policy and Practice Information Coordinating Centre systematic mixed-method approach was adapted to locate, appraise, extract and synthesise evidence. We used Ley's cognitive hypothetical model of communication and subsequently developed a theoretical framework explaining information exchange in healthcare contexts.

**Results:**

Young people and parents believed that healthcare professionals were only interested in medical management. Young people felt that discussions about their epilepsy primarily occurred between professionals and parents. Epilepsy information that young people obtained from parents or from their own efforts increased the risk of epilepsy misconceptions. Accurate epilepsy knowledge aided psychosocial adjustment. There is some evidence that interventions, when delivered in a structured psycho-educational, age appropriate way, increased young people's epilepsy knowledge, with positive trend to improving quality of life. We used mainly qualitative and mixed-method evidence to develop a theoretical framework explaining information exchange in clinical encounters.

**Conclusions:**

There is a paucity of evidence reporting effective interventions, and the most effective ways of delivering information/education in healthcare contexts. No studies indicated if improvement was sustained over time and whether increased knowledge was effective in improving in self-care. Current models of facilitating information exchange and self-care around transition are not working well. There is an urgent need for further studies to develop and evaluate interventions to facilitate successful information exchange, and follow young people over time to see if interventions showing early promise are effective in the medium to long-term.

## Background

Epilepsy is a common long-term neurological condition associated with abnormal brain function and seizures [1]. There are approximately 38 different types of seizures and 30 epilepsy syndromes [2]. The majority of epilepsy syndromes commence in childhood and/or adolescence [3]. It is important that the type of seizure and epilepsy is identified and classified in order for healthcare professionals, especially epilepsy nurses, to facilitate ongoing child and family education to optimise long-term management, and to promote self-care for young people and appropriate healthy lifestyle choices [4].

Forsgren [5] estimated an age-specific global incidence of 3.5 million people developing epilepsy on an annual basis, 40% are children under 15 years old, 40% young people and adults aged 15-65 years and 20% are elderly. Epilepsy incidence in childhood is higher than in adulthood [6,7]. Approximate 700 per 100,000 children under the age of 16 years have epilepsy in comparison to 330 per 100,000 in adults [8].

### Challenges to information exchange and nurturing self-care expertise in young people with epilepsy, and parents

Within the context of this review we have defined information and knowledge exchange as the active or passive process of exchanging or imparting information, knowledge and skills between healthcare professional and young people in routine clinic or healthcare encounters. Healthcare professionals and young people exchange information and impart knowledge and skills in a variety of ways, which may include demonstrating, explaining, monitoring and feeding back, and using or referring to a variety of information resources and materials (e.g. books, leaflets, internet sites etc), and referral to epilepsy charities and support groups for additional information and support.

Epilepsy specific, biological and psycho-social factors act as barriers and enablers to information exchange and nurturing of self-care practices in healthcare contexts. Elliott et al [9] identified that the intrusive impact of experiencing seizures affected all aspects of children and young people's lives. Despite 63% stating they were happy most of the time the unpredictability of their seizures caused the majority to experience periods of intense emotional distresses. Other feelings included worry or fear (49%), sadness, dysphoria or depression (45%) and anger/frustration (67%).

When young people continue to experience seizures despite anti-epileptic drug treatment, they are more likely to be affected by other co-morbidities. Common co-morbidities include, learning impairment due to brain malformation, depression or social maladjustment due to seizures, cognitive impairment due to their anti-epileptic drug treatment, behavioural problems, and difficulty sleeping [10].

Despite not having an associated disability all children and young people with epilepsy are at risk of behavioural and learning difficulties [11,12]. Young people with epilepsy may limit disclosure of their condition, may not accept epilepsy as a long-term condition and may not take their medication as prescribed leading to increased risk of physical injury due to seizures [13]. Parents reported that their child with epilepsy was negatively affected by stigma, behaviour at school, and memory/concentration problems. Whereas young people themselves in the same study did not report similar issues and were perceived to try to deny their problems [14].

Current philosophies of self-care and long-term management of chronic diseases focus on young people becoming expert in their own care by the time they transition to adult services. Adults, whose epilepsy began in childhood, have however identified important gaps in communication during their early years. Consultation about *their *epilepsy was discussed with *their *parents, with little or no information on self-care, which they believe has resulted in current poor self-management and psychosocial problems [15]. Younger children usually depend on their parents for explaining concepts of epileptic phenomena and their needs are frequently defined from the perspective of professionals [16].

The time (age 13-19 years) around transition to adult services is known to be a particularly challenging time for young people generally [17]. Support and understanding from a parent is invaluable in helping a young person develop life skills and confidence in managing and living with epilepsy. However, Freeman et al [18] found that parental overprotection and restriction of young people socially led to high levels of anxiety and lacking in confidence.

The time building up to and during transition of young people with epilepsy from children's to adult healthcare services is particularly important. Little is known about their specific experiences of information needs and knowledge exchange in clinical contexts at this time.

## Methods

The following objectives were developed to help organise the search and synthesis of evidence:

a) To determine what is known to be effective in delivering information/education to young people with epilepsy and their parents.

b) To explore what mixed-method evidence tells us about knowledge and understanding, use of information, information needs and experiences of young people aged between 13-19 years of age with epilepsy, and their parents, in health care contexts, and

c) To describe the facilitators and barriers to information exchange in health care contexts with this group of young people, and their parents.

In considering objective b, we were aware that exploration of evidence was likely to be multi-layered and complex. In considering how to interrogate the evidence and interpret findings in light of gold standard epilepsy management, we were interested to see what evidence told us about:

• What young people know about their epilepsy?

• What do they need to know about their epilepsy?

• What do young people not know about their epilepsy and why?

• What positive and negative impacts does appropriate knowledge and understanding have?

### Study design

**Figure 1:**
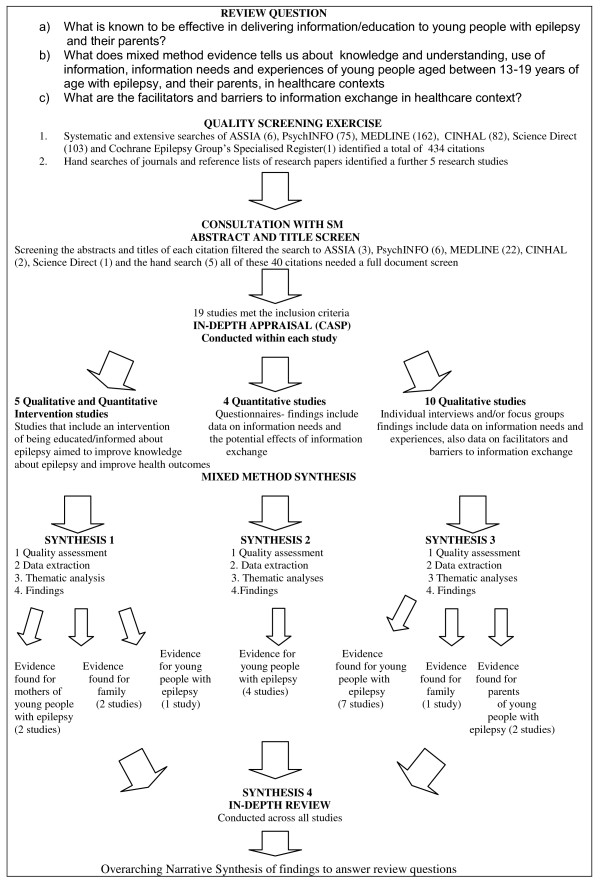
**Flow diagram of the review process**.

As it was likely that mixed method evidence would be required to address the review objectives, a mixed-method systematic review design based on the Evidence for Policy and Practice Information and Co-ordinating (EPPI) Centre [19] and the EPPI Centre Guidance on synthesis of mixed-method evidence by Oliver et al 2005 [20], was selected. The model by Oliver et al 2005 [20] was adapted to enable quality screening and synthesis of evidence within three separate synthesis streams (see Figure 1). Evidence was initially organised and synthesised by study type into three streams (intervention, other quantitative, and qualitative).

The synthesis of intervention studies was designed to address objective a, the synthesis of other quantitative and qualitative studies (streams 2 and 3) was designed to explore objectives b and c.

### Data analysis

**Figure 2:**
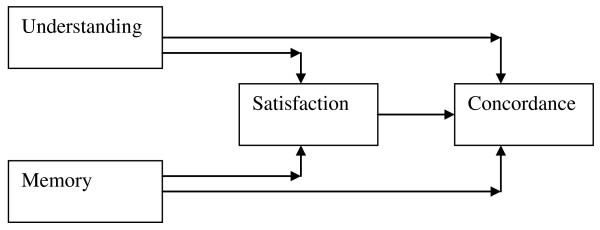
**Adapted Ley's cognitive hypothesis model of communication**.

Randomised controlled trials reporting similar interventions with common outcome measures were not located so it was not possible to perform a meta-analysis. Therefore, tools and techniques from the narrative synthesis toolbox [21] were used to synthesise evidence from the three streams, and in an overarching fourth narrative synthesis. Synthesising evidence within the three streams involved thematic analysis and the approach described by Thomas and Harden 2008 [22] was adapted for this purpose. All findings were entered into Nvivo 8 computer software [23] and the synthesis commenced with line by line coding and then inductive coding from the text to capture meaning. Within a bio-psychosocial context we used Ley's 1988 [24] cognitive hypothesis model of communication (see Figure 2) to inform interpretation of evidence.

### Search strategy

A simple search strategy as advocated by Flemming and Briggs 2007 [25] was used to locate studies and is summarised in the SPICE Table three [26], defining the Setting, Perspective, Interventions, Comparisons, Evaluations and Methodological approaches. The search strategy was developed with key concepts of interest from the objectives. The search terms used included the recognised Medical Subject Heading (MESH) terms and non-MESH. The search terms used included adolescence (adolescent*), young person or teenager, aged between 13-19 years, combined with epilepsy or epilepsy service and parent or family, information or information needs, knowledge or health knowledge, education or educational needs and transition.

**Table 1:** SPICE search strategy

Setting	Perspective	Intervention	Comparison	Evaluation	Methodological approach
Information and knowledge exchange of young people with epilepsy age 13 to 19 years old, and their parents, in healthcare contexts	Evidence of effectiveness of interventions Views, experiences and perceptions of young people and parents	Any interventions	Controlled intervention studies, before and after studies, intervention studies with no control, validation studies with or without control Qualitative comparison of views, experiences and perceptions of young people and parents	Comparison of outcomes to determine effectiveness Comparative and thematic analysis of qualitative evidence	Quantitative Qualitative Mixed method

SAL and SM conducted an electronic search of the Cochrane Epilepsy Group Specialised Register and The Cochrane Central Register of Controlled Trials (January 2010), ASSIA (earliest-2010), CINHAL (1980-2010), MEDLINE (CSA, earliest-2010), PsychINFO (CSA, earliest-2010), Science Direct (full text e journal database) and the Database of Abstract of Reviews of Effectiveness (DARE). We supplemented electronic searches with hand searching of key epilepsy journals Seizure, Epilepsia, Epilepsy & Behaviour, and ancestral searching of reference lists from relevant studies. We included studies published in English and English language translations. Types of studies included mixed-method intervention and non-intervention studies, randomised control trials (RCT) (before and after studies) involving young people with epilepsy and/or parents of young people with epilepsy. RCT and intervention studies were included within the final review if they identified the knowledge and/or information base of the participants about epilepsy then provided an intervention such as education, or giving information about epilepsy (oral and/or written) and then evaluated the effect of the intervention, such as improved knowledge about epilepsy or improved health outcome. Non-intervention studies such as quantitative and qualitative studies were included if they broadly reported young people and/or parent perspective on their information needs. Studies that included a wider age group than age 13-19 years were only included if data for young people aged between 13-19 years of age could be extracted separately.

### Study selection

The initial electronic search identified 434 citations. From these citations the titles and abstracts were reviewed, of which 40 citations required a full document screen to determine if they met the inclusion criteria. It was unclear whether these studies targeted children, young people and/or adults. Hand searching of key epilepsy journals and reference lists identified 5 further studies that required a full document screen. Nineteen out of 40 studies met the inclusion criteria and were included in this review.

### Quality Assessment

Studies were appraised within each stream separately using the relevant versions of the Critical Appraisal Skills Programme tool CASP [27]. None of the 19 included studies were excluded although there were variations in the quality of reporting. Ten corresponding authors were contacted by e-mail for additional information and some responded with further information. No study had a fatal flaw (the threshold for exclusion).

### Data extraction and management

SAL extracted and summarised data in tables and templates adapted from National Institute for Health and Clinical Excellence NICE guidance [29]. Streamed and extracted data are summarised in Tables 2, 3 and 4. JN checked data extraction and any queries were resolved by consensus with SAL. Streams of extracted evidence were then analysed thematically as described in the methods section. We also developed a set of propositions to explore further in a subsequent comparative qualitative case study of young people with epilepsy undergoing transition through different service models from child to adult service provision. A proposition is an idea, concept or statement with inherent meaning and we have reported the propositions as an integral component to the narrative and thematic analysis. Propositions were then used as the major building blocks in the construction of the analytical model and theoretical framework.

**Table 2:** Summary table of included Intervention studies

References	Study type/Intervention	Participants	Setting/context	Outcomes	Results	Methods/Quality
Shore et al 2008 [32]	Pre and post Intervention study *Content*: Seizure and Epilepsy Education (SEE) program- 1^st ^day education about epilepsy, seizures, AED & lifestyle management 2^nd ^day- psychosocial, coping skills, education and employment (n = 17 families) *Duration*: 2 consecutive weekends days 8 hours per day *Delivered by*: Robert Mittan who designed the original SEE program for adults	Young people aged 13-18 years old 11 young people 7 boys 4 girls 13 families in total completed the whole study Caucasian African-American	Not stated	Follow up data was obtained at Baseline child n = 9 Parent n = 16 1 month child n = 8 parent n = 14 6 months child n = 9 parent n = 16	1. Parent's demonstrated improved knowledge at 1-month and 6-months (adjusted p values = 0.001 and <0.001, respectively 2. Parent's less emotional impact at 6-months (adjusted p value = 0.033) No significant change to young people knowledge about epilepsy (0.05 p level significance adopted) Internal consistency Cronbach's α ranging from 0.74 to 0.97	Met all criteria however: Parent data strong Young person data weak Intervention not appropriate to the developmental and educational level of the young person 5 families dropped out No follow-up data for 3 young people 1 parent did not complete 1-month follow up

Vona et al 2009 [33]	Pre and post Intervention study *Content*: To read a Brochure (English n = 20 and Spanish n = 20) with 6 subsections relating to co morbidities associated with epilepsy N = 40 *Duration*: Time it took to read complete questionnaires and read Brochure *Delivered by*: Authors of study	20 Hispanic mothers 20 Caucasian mothers	Clinic waiting room	Post intervention questionnaire was compared to the pre intervention questionnaire	1. Mothers demonstrated significantly increased knowledge about co morbidities (F(1.38 = 10.84, p < 0.002) 2. greater knowledge about effective mental health care (F(1.36) = 3.80, p < 0.06) no significant effect in between mother groups (0.05 p level significance adopted)	Questionnaires and Brochure not previously validated Due to recruitment strategy no data on non responders No demographic data on participants

Buelow 2007 [34]	Feasibility Study *Content:* Day 1- Introduction and giving information about epilepsy Day 2 & 3- learning advocacy skills Day 4- teaching parents how to influence policy n = 4 *Duration*: 4 days *Delivered by*: The author and one parent expert	4 mothers	Not stated	Open-ended questions to the group at the end of each day, the response data collected and qualitatively analysed	Lifestyle changes-mothers gained knowledge and skills on how they can take control and plan their child's transition and dealing with health, social care and education Thematic analyses	Recruitment strategy weak Intervention validated by conducting a pilot study and focus group with experts

Jantzen et al 2009 [30]	Pre and post Intervention study 2 day course (14 hour per course) or 2.5 days (16 hour per course) Questionnaires: Parents-Epilepsy Knowledge Profile (EKP-G) 55 true/false items (34 medical knowledge and 21 social knowledge) Children's-modified EKP 27 true/false items medical and social Parent and child questionnaire on knowledge-Internal consistency coefficient of the scale was α = 0.58 in the study sample	44 young people aged 12-16 years old 72 parents (21 children) control group 31 children, 39 young people and 72 parents	Not stated	Pre intervention questionnaire and 6 months post intervention questionnaire Waiting time control group 6 months prior intervention and just before the intervention	Young people increased medical knowledge (MK) and seizure triggers (ST) post intervention Mean (SD) MK: Baseline 19.52 (4.42) Post 24.91 (3.57) ST: Baseline 8.18 (2.46) Post 9.50 (2.47) Parents increased knowledge on medical and social aspects of epilepsy MK: Baseline 27.54 (3.72) Post 29.83 (2.51) ST: Baseline 12.28 (2.41) Post 14.97 (2.16)	Control group matched Follow up assessment Well researched prior to setting intervention Piloted by children, young people and parents to validate intervention

Snead et al 2004 [31]	Pre and post Intervention study One hour a week for six weeks, didactic session then follow a group discussion and use of audio visual media and handouts	1^st ^group total 7 3 boys 4 girls	Neurology department	Pre and post intervention Questionnaire delivered just before intervention and 6 weeks later	Positive trend towards improvement in quality of life Statistical analyses conducted using a paired *t *test and a nonparametric χ^2 ^test.	Researchers trained in neuropsychology And working with young people Intervention was piloted and amended following feedback from participants to increase validity and reliability

**Table 3:** Summary table of included Quantitative studies

Reference	Study Design	Research Question	Setting Context	Main Results	Methods/Quality	Other notes
Baker et al 2005 [35]	Quantitative-Matched, controlled study by means of a number of questionnaires 6 questionnaires for all young people: 1. The Rosenberg self-esteem scale 2. The social avoidance and distress scale (SADS) 3. The Birleson Depression scale (BDS) 4. The Leyton Obsessional Inventory (LOI)- child version 5. The Children's Depression Inventory (CDI) 6. The Schonell Reading Test Additional 2 questionnaires for young people with epilepsy with epilepsy 7. The impact of epilepsy scale 8. Adolescents knowledge of epilepsy questionnaire n = 75 aged 12-18 years	To investigate the psychological and social impact of epilepsy on young people and to identify to what degree clinical and demographic variables and knowledge of epilepsy could influence psychological functioning	Epilepsy centres UK	Young people with epilepsy who had more epilepsy knowledge were less depressed p = 0.039 mean 7(5-9 confidence interval) they also had higher level self esteem p <0.026 mean 33(31-34 confidence interval) and low social anxiety p = 0.039 mean 7(5-9 confidence interval) Young people with epilepsy who had low Epilepsy Knowledge were more depressed p = 0.039 and had low self esteem p = 0.026 mean 11(9-14 confidence interval) and mean 28(27-31 confidence interval) respectively Mean (95% confidence interval) level of knowledge of epilepsy	Validated questionnaires Control group Participants from a specialist centre	

Kongsaktrakul et al 2006 [43]	A Questionnaire conducted in the epilepsy clinic adopting the following sequence: The personal Data Form, the self-care behavioural scale, The Epilepsy Knowledge Scale, The Epilepsy Self-Efficacy Scale, the Family APGAR Questionnaire, and the Friend APGAR Questionnaire n = 121 aged 14-21 years	To determine a causal relationship among age, family income, support, epilepsy knowledge, epilepsy self-efficacy and self-care behaviour among young people with epilepsy	Clinics Thailand	Young people with epilepsy showed: Improved self-care behaviour p = <0.001 Positive direct effect self-efficacy p = <0.05 Family income positive effect p <0.05 CFI 0.99	Cross sectional design Participants from specialist centres	

Bell et al 2002 [46]	20 page postal Questionnaire commissioned by Department of Health Clinical Standards Advisory Group (CSAG) about services for people with epilepsy n = 795 of which: (n = 13-16) 16-17 years old (n = 20-21) 18-19 years old (n = 29-30)	To establish whether women with epilepsy recall being given information on topics relating to childbearing	Home UK	31% (5 out of 16) young girls aged 14-15 years received information about the interaction between their Anti-epileptic drug treatment and the oral Contraceptive pill. 20-35% from 14-17 years and 55%-65% aged In-between 16-19 years received information. Teratogenesis of AED	Data could have been better displayed for age ranges	

Hirfanoglu et al 2009 [43]	Questionnaire 46 items for children 43 items for parents n = 220 children n = 77 parents	To evaluate knowledge, perception and attitude towards epilepsy and how this correlates with quality of life and stigma among children with epilepsy and their families	Clinics Turkey	Adolescents: increased epilepsy knowledge compared to younger children (p = 0.0001, r = 0.294) increased stigma (p = 0.0001, r = 0.256), depression p = 0.0001, r = 0.276) longer duration of seizures equated to negative attitude towards epilepsy p = 0.001, r = 0.223) Parents- 20% did not inform their children about epilepsy, 42% did not know what to do during a seizure	Researchers differentiated between children and adolescents and demonstrated statistical significance for adolescents in knowledge, stigma and depression	

**Table 4:** Summary table of included Qualitative studies

Author and date	Study design And Research type	Research Question	Age range of young people, sample size, context and Country	Main Result
Admi H and Shaham B 2009 [39]	Qualitative life history method via in-depth interviews	Exploration of life experiences of young people with epilepsy	15-24 years old 11 girls and 3 boys In clinic or outside hospital Northern Israel	Younger adolescents did not want information Older adolescent wanted more

McEwan et al 2004 [40]	6 Focus Groups	Describe Quality of Life in young people with epilepsy	12-18 years old 6 boys 16 girls Neuroscience Unit UK	Younger adolescent needed more information than older adolescent Reluctance to ask questions due to fear of consequences. Lack of knowledge about epilepsy related issues Inaccurate knowledge of legislation Wanting accurate information

Eklund and Sivberg 2003 [41]	Qualitative Individual interviews	Describe lived experience of young people with epilepsy and their coping skills	13-19 years old 3 boy 10 girls Held at Paediatric Department Sweden	Misconceptions about epilepsy. They did not know if being tired, problems sleeping, difficulty concentrating and memory impairment was due to their epilepsy or taking medication. Barriers in effective communication to information exchange with doctors

Kyngas 2003 [42]	Qualitative Individual interviews	Describe patient education from young persons perspective	13-17 years old 24 girls 16 boys (8 young people had epilepsy) Held at Hospital or child's home Finland	Young people wanted more practical information, frequent educational sessions and opportunity to ask questions

Beresford and Sloper 2003 [37]	Qualitative study be means of one-to-one interviews and focus groups	To explore the experiences of chronically ill young people in communicating with health professionals, including the identification of factors which hinder or facilitate their use of healthcare professionals as an information source	10-16 years old 36 girl 27 boys (10 young people had epilepsy) Interviews held at child's home Focus groups near to child's home UK	Barrier to communication-different doctors, limited time to talk and too many other healthcare professionals in the clinical room inhibited discussing personal issues. Presence of a parent can be both inhibitive and supportive. Lacking confidence to initiate communication. The young people felt they did not know how to ask the question. Reluctance to ask questions that may results in negative consequence

Wilde and Haslam 1996 [38]	Qualitative study semi-structured interviews	To explore the issues affecting young people with fairly significant epilepsy	13-25 years old 15 girls 9 boys Held at Hospital clinic UK	Barriers to information exchange concentrating on medical aspects rather than giving practical advice living with epilepsy. Lack of continuity and not developing a professional relationship with those managing their epilepsy due to constant change of personnel

Sanger et al 1993 [47]	Structured Individual interviews	To identify developmental sequences in children understanding of the cause of their seizure disorders	5-16 years old 19 boys 31 girls Site not stated USA	Misconceptions about epilepsy

Buelow et al 2006 [4]	Individual Interviews	To identify sources of stress of parents of children with epilepsy and intellectual disability	Children and adolescents (9-16 years, 7 boys/13 girls 20 parents-18 mothers, 1 father and 1 step-father Site not stated USA	Lack of information about their child's epilepsy They perceived the doctor's focus was on medication management and number of seizures and did not listen to parent concern The stress of the child's epilepsy affected family relationships, Transition skills not addressed at schools

McNelis et al 2007 [44]	2 Focus Groups Children and Young People 2 Focus Groups parents	In-depth exploration of concerns and needs of children with epilepsy and their parents	1^st ^group had 6 children (7-14 years old, 3 girls and 3 boys) 2^nd ^group had 5 children (9-15 years old, 2 girls and 3 boys) 1^st ^parent group 7 (6 mothers 1 father) 2^nd ^parent group 8 (6 mothers and 2 fathers) Community setting USA	Barriers to information exchange Young people: wanted to be equally informed as their parent's and discussion at their level They felt ignored by the doctor and discussion about them occurred with the parent. Their own basic questions not being addressed. Lack of information the children develop misconceptions Inability to ask questions, The need to have continuing information and new knowledge to 'keep abreast' of the epilepsy.

Swarztrauber et al 2003 [48]	1 focus group with 4 adolescents and 1 focus group with 4 parents	To understand patient attitudes about the treatment of medically intractable epilepsy	Young people were aged 13, 14, 16 and 17 years old Held at University of California USA	Parents not receiving adequate information from physicians They did not receive sufficient information about anti-epileptic drugs and their side-effects Despite seeing a doctor, the young people obtained their information from their parents

### Interpreting the entire dataset and developing a theoretical framework

**Figure 3:**
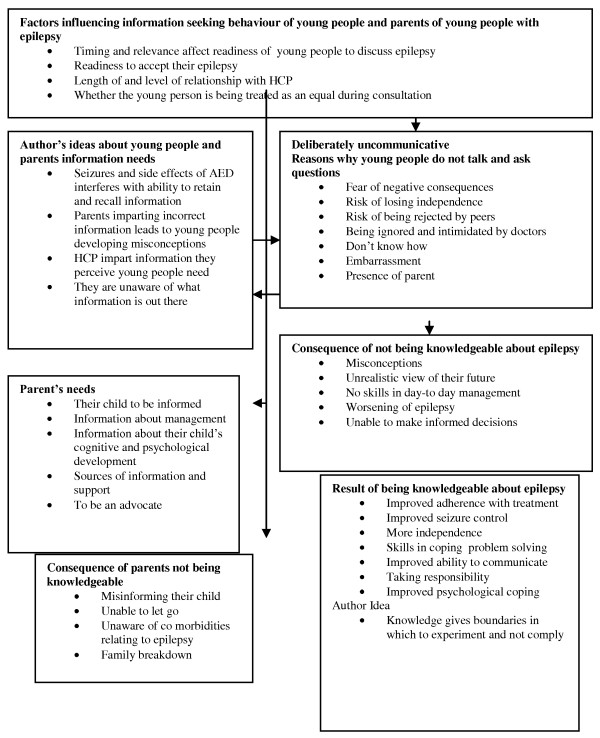
**Analytical model of factors influencing information exchange in healthcare contexts**.

**Figure 4:**
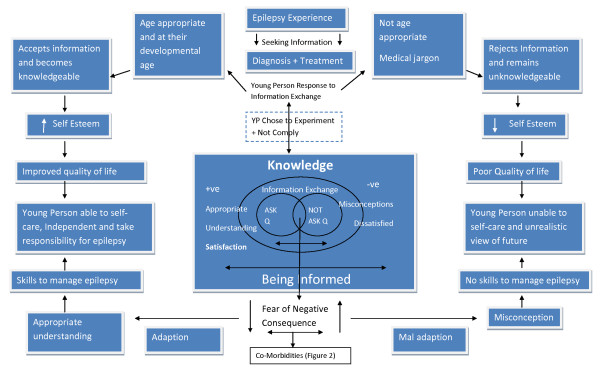
**Theoretical Framework of the positive and negative influences of knowledge exchange in healthcare context**.

**Figure 5:**
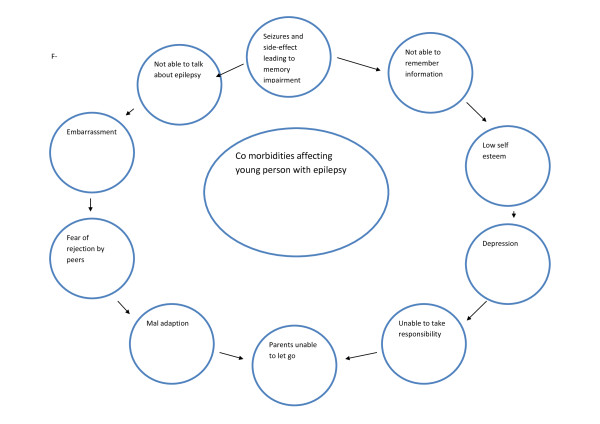
**Co-morbidities experienced as a result of knowledge exchange**.

SAL and JN then adapted procedures described by Pound et al [49] for synthesising, exploring, further mapping, integrating propositions, interrogating and understanding findings from all phases, and incorporating our expert knowledge. We spent time developing an initial analytical model of factors influencing information exchange in healthcare contexts (see Figure 3). During several subsequent meetings over nine months, SAL and JN developed and refined the analytical model to help understand knowledge exchange, retention, use and impact in young people with epilepsy, and over time developed new theory, which is presented as a new theoretical framework to inform the discipline and science (see Figure 4 and 5).

## Results

Synthesis of intervention studies (stream 1).

We synthesised intervention studies to determine what is known to be effective in delivering information/education to young people with epilepsy and their parents?

We found a paucity of evidence evaluating interventions. We synthesised n = 5 studies (see Table 2) and developed 3 propositions. In the following section we present each proposition followed by a summary of findings from which propositions were developed.

### Proposition: Age appropriate psycho-educational programmes for young people with epilepsy show potential in increasing medical knowledge and improvement in health related quality of life

Although there were few studies, they show that early developments in structured age-appropriate educational programmes for young people have demonstrated positive trends towards improvement in medical knowledge [30] and health related quality of life [31].

### Proposition: Being educated and being knowledgeable about epilepsy empowers parents to be an advocate for their child

Studies indicated that an educational session improved the knowledge of parents and 6 months after the session parents reported fewer unmet needs, were less worried and more confident in managing seizures [32]. Effect of the intervention included parents feeling less emotional impact of their child's epilepsy [33]. Frequent educational meetings enabled parents to understand issues in all key areas surrounding epilepsy and to develop an action plan for their child to use in partnership with health, education and social services, thereby potentially leading to their child becoming independent [34].

### Proposition: Being educated about epilepsy makes parents realise what knowledge they do not possess and motivates them to seek more information

We found that mothers of children with epilepsy were not aware that their children's problems were linked with epilepsy. Mothers demonstrated increased knowledge about their child's behaviour and cognitive co-morbidities of epilepsy after reading a brochure and they asked for more information about epilepsy and other co-morbidities [33]. Attending educational meetings caused mothers to realise that they did not possess the knowledge and skills to help their children, and being educated over time enabled them to develop problem solving skills they did not have before [34].

### Synthesis of quantitative and qualitative studies (streams 2 and 3)

We synthesised separately then together 4 quantitative and 10 qualitative studies (see Tables 3 and 4) to ascertain what evidence told us about knowledge and understanding, use of information, information needs and experiences of young people aged between 13-19 years of age with epilepsy, and their parents, in healthcare contexts. We also wanted to identify barriers and facilitators to information exchange. From using thematic analysis to synthesise and understand evidence, we also developed 12 propositions from synthesised evidence.

### Proposition: Young people need accurate information about epilepsy to aid psychosocial adjustment

Baker et al [35] and Kongsaktrakul et al [36] found that the more knowledgeable young people were about their epilepsy the more positive were their health outcomes. Low level of epilepsy knowledge was found to be associated with higher levels of depression, lower levels of self-esteem and higher levels of social anxiety. Psychosocial impact of epilepsy appeared to revolve around social aspects and manifested in higher levels of social anxiety. The importance of epilepsy knowledge appeared to be vital to their psychosocial adjustment. Epilepsy self-efficacy and epilepsy knowledge resulted in a positive effect on self-care behaviour and knowledge of epilepsy had a positive effect on self-efficacy [35]. Therefore being informed enabled the young people to take better care of their condition.

### *Proposition*: Young people need practical advice about lifestyle management but think that healthcare professionals are only interested in medical management of epilepsy

Young people perceived that healthcare professionals were only interested in medical aspects of their condition [37]. Inadequate explanation about the diagnosis was given by doctors in clinic and communication concentrated on medical aspects rather than giving practical advice on living with epilepsy [38]. Younger people (age 13-15 years) showed less desire to know about the cause for their epilepsy and wanted more information on the 'here and now', whereas the older the young person (16-19 years) wanted to know about the future including education, employment, marriage and having children [39].

Young people wanted accurate information and help on realistic management of seizures [40]. They also did not know if being tired, having problems sleeping, difficulty concentrating and memory impairment were due to their epilepsy or taking medication [41]. Those young people who were more knowledgeable in identifying triggers for seizures, understood the importance of taking medication were able to take control of their epilepsy and maintain their own safety [39]. Young people recognised that the more practical skills and knowledge they possessed about epilepsy the more independent they could become [42]. A third of young people experienced lack of support from healthcare professionals [43]. Ley's [24] model hypothesised that dissatisfaction with consultation correlated with poor recall and understanding of information.

### *Proposition*: Parents need practical advice but think that healthcare professionals are only interested in medical management of epilepsy

Findings showed that parents needed information about their children's epilepsy and other lifestyle factors in order to make informed decisions. However they do not want to be overwhelmed with information [44]. Parents spoke of their concern about the lack of information about their child's epilepsy. They perceived the doctor's focus was on medication management and number of seizures and did not listen to parent concerns about side effects. Parents needed far more information about prognosis, managing problems at school and their child's behaviour.

The stress of their child's epilepsy affected family relationships caused poor communication within the family. In one study one couple divorced as a result, some parents were unable to go to work and unable to give time to other siblings [45]. Parents needed a timely contact point to obtain information as to what to do when their child was in a seizure [44].

### *Proposition*: Young people do not receive the right information in the right frequency and at the right time during their teenage years

Young people wanted individualised structured education at a variety of intervals dependent on need throughout their teenage years and not all at once at the time of their diagnosis. Admi and Shaham [39] showed that the information young people needed was diverse, dependent on age, and what seemed appropriate and important to them at different stages in their development varied over time. Young people described lack of information given in clinic and their inability to discuss sensitive issues with a doctor [40].

### *Proposition*: Young women are not consistently receiving or remembering gender specific advice

We found that young women wanted information about their medication and its affect on menstruation and fertility [41] as well as having children [39,40]. Bell et al's, [46] postal questionnaire reported that 31% (5/16) young women aged 14-15 years old remembered receiving information about the interaction between their anti-epileptic drug treatment and the oral contraceptive pill. Approximately 55%-65% of the older girls received this information, and 20-35% (age range 14-17 years) remembered receiving information about teratogenesis.

When young people experienced seizures at the time of being given information they had difficulty in recalling what was said [41]. Cognitive difficulties due to seizures and/or side effects of medication can also cause young people to have difficulty in concentrating and remembering [38].

### Proposition: Misinformation leads to misconceptions and uncertainty about epilepsy, and inability to cope with stigma

Young people made improbable links and tried to make sense of the epilepsy through personal experiences rather than actual facts about the condition [43,47]. Misconceptions about seizures due to lack of information raised fears such as risk of dying and becoming disabled [44]. Some young people had been misinformed that they may grow out of their epilepsy and were disappointed that they had not [41]. Lacking of epilepsy knowledge generated 'distortions and misconceptions' leading to heightened stigma surrounding epilepsy [35] and felt stigma from their friends [43]. Young people felt that they were being treated differently due to the lack of knowledge about epilepsy in society [39,41]. Seventy-one percent of young people in one study reported negative reactions towards them, including prejudice and discrimination sourced from secondary school [38]. Teasing, name calling and bullying caused great distress. Very few were able to inform teachers about their epilepsy and therefore suffered in silence. This enacted stigma dramatically reduced once young people had left school [38].

### Proposition: To be able to self-care and be independent of their parents, young people realise they need to know more about epilepsy to take responsibility

Young people saw that their parents gave them more independence if they knew more about epilepsy [40]. They realised the more knowledge they had leads to independent living and they expected healthcare professionals to teach them the necessary skills to solve their own problems [42]. Unfortunately, they perceived that in healthcare context, medical management was provided at the expense of being given practical information [38].

### Proposition: Young people do not know HOW to ask questions about their epilepsy

Young people felt they did not know how to ask questions. Their need for personal or sensitive information was itself a barrier preventing using healthcare professionals as a source of information. There was a reluctance to ask a question in-case it resulted in negative consequences, such as having to be hospitalised if they revealed worsening of symptoms. Young people feared asking a question which may reveal that they are not taking their medication [37]. Young people were also reluctant to ask questions about alcohol and pregnancy due to fear that it would imply that they drank too much were having under age sex [40]. Some young people wanted information about activities which they may not be allowed to do, and they may not to ask questions about these activities due to the risk of losing independence [37]. When healthcare professionals facilitate discussion at their level, young people had confidence to express themselves [42].

### Proposition: The clinical encounter mainly acts as a barrier to information exchange

Features of the clinical encounter such as seeing a different doctor every clinic visit was a big barrier to communication. Limited time to talk and too many other healthcare professionals in the clinical room inhibited the discussion of personal issues. Due to the high status of a doctor some young people lacked confidence to initiate communication [37,41]. Having had a negative experience in the clinic room and their appointment being rushed left no time to build a rapport resulting in a negative encounter [42]. Professionals with appropriate communication skills and techniques, and a quiet room with no distractions were found to facilitate more opportunity for questioning and knowledge exchange [42]. The presence of a parent can have both positive and negative impacts on knowledge exchange. Presence of some parents inhibited the young person raising private and sensitive issues, whereas the presence of a parent can also be supportive and boosted the young person's confidence [37,41].

### Proposition: Healthcare professionals lack facilitative skills of working in partnership with young people, with or without their parent present

Young people felt healthcare professionals excluded them from discussion surrounding their epilepsy. The discussion focused on the parent and young people did not understand what was said due to medical jargon [41]. Young people wanted to be as equally informed as their parents and have discussion at their level [44]. Despite physically being in clinic the young people often obtained their information from their parents at a later stage [48]. When young people had difficulty understanding information given to them they lost interest in the consultation and stopped listening [42] and they adopted a passive role [44].

Healthcare professionals were also said not to be perceptive to the unspoken concerns of the young people [37]. Young people had difficultly expressing their feelings. Their interpretation was that healthcare professionals were well aware that they had emotional issues to discuss but they pretended not to be aware of it to avoid discussion as they did not know how to deal with it. Young people wanted healthcare professionals to be knowledgeable about their developmental stages and be responsive to their needs [42].

### Proposition: Lack of effective partnerships and interruptions to continuity of care are having a detrimental effect on information exchange and knowledge use by young people

Not being a partner in their own care, lack of continuity, and not developing a professional relationship with those managing their epilepsy due to constant change of personnel negatively affected young people [38]. Due to continual change of healthcare professionals young people felt that they had to repeat their history and go over the same issues all over again leading to them not being able to progress [37]. The length and positive nature of the relationship with healthcare professional was an important success factor that enabled trust to develop, and the longer the relationship the more confidant young people were in asking questions [37,42].

### Proposition: Parents are unaware of what epilepsy knowledge they do not have

Some parents did not know what epilepsy knowledge they did not have and therefore need to have continuing information and new knowledge to keep abreast of their child's epilepsy. Parents were frustrated about not receiving adequate information from healthcare professionals and had to seek their own information from the internet [44].

### Overarching narrative synthesis of the entire dataset and development of the analytical model and subsequent theoretical framework

There is little evidence of effectiveness concerning what works, so this overarching narrative synthesis focused on moving beyond the thematic analysis and theoretical propositions to mapping ideas and generating and interrogating relationships in the synthesised body of evidence to develop a theoretical framework of the positive and negative influences of accurate and appropriate knowledge exchange and use of knowledge by young people with epilepsy. The analytical model of factors influencing information exchange in healthcare contexts is outlined in Figure 3 and then used this model as a basis for developing a theoretical framework (see Figure 4 and 5).

The logic of the theoretical framework is described in the following paragraphs. Evidence suggests that young people with epilepsy who are receptive to their diagnosis, ready to accept age appropriate information, and able to retain and use information are better able to engage in the consultation with healthcare professional and ask questions. If they receive high quality accurate information in appropriate formats that is actively facilitated in an individually-tailored and young person-centred way then young people are more likely to develop knowledge and skills to manage their epilepsy. As a consequence of being receptive and taking responsibility, young people are more likely to be afforded freedom to make their own decisions by their parents and they are more likely to be able to lead an independent life.

Whereas, young people with epilepsy, who are not receptive to their diagnosis, are not ready to accept information, are not as able to use and retain information. Young people who do not interact and ask questions during their consultation, develop misconceptions, are afraid to ask questions due to fear of negative consequences, are more likely not to be afforded freedom to make their own decisions by their parents and they are more likely not to be able to lead an independent life.

It is also likely that for a variety of complex reasons there are disconfirming cases of young people with epilepsy who chose not to and/or are unable to follow the path towards autonomy and manage their epilepsy effectively despite being informed about their epilepsy and the theoretic framework also includes consideration of outcomes that are influenced by biological, psychological and/or social factors associated with epilepsy.

With clinicians in mind, we have also summarised in Table 5 the critical success factors for information exchange in clinic contexts for young people with epilepsy aged 13-19 years (the time period when young people are prepared for and transition from children's to adult health services).

**Table 5:** Critical success factors for information and knowledge exchange in clinic contexts for young people with epilepsy during transition aged 13-19 years.

• Availability of accessible, age and gender appropriate epilepsy information on a variety of self-care and lifestyle management issues • Provision of information in a variety of types and age-appropriate formats likely to attract and engage young people • Awareness of all factors (e.g. biological etc) that act as facilitators and barriers to information exchange • Active facilitation by healthcare professionals of practical advice and information resources about daily lifestyle management - including sensitive topics • Introduce and facilitate information in clinical encounters at staged and regular intervals throughout teenage years • Active ongoing engagement and follow up by healthcare professionals with young people to ascertain recall and understanding of the information given • Age-appropriate and individually-tailored facilitation and discussion with young people to encourage them to ask questions • Actively building rapport by ensuring the same healthcare professionals at clinical encounters • Awareness that some healthcare professionals are themselves a barrier to positive information exchange as they are unable to relate to young people in age-appropriate ways • Provide opportunities for young people to talk openly without parental presence • Parents need consistent and ongoing epilepsy information, practical advice and high levels of support from healthcare professionals to enable their child to safe self-care and become independent • Awareness that some young people will need ongoing support and high levels of repeated information in order to take on some or all of their self-care • Engagement with young people to inform service delivery and organisation of care (e.g. length and frequency and clinic consultation and other modes of follow up). • Regular and meaningful review of the effectiveness of service provision and strategies to promote independence and self-care with involvement of young people as service users. • Epilepsy charities produce a wealth of information, which appears to be under utilised by healthcare professionals

## Discussion

The major outputs from the review are a new analytical model of factors influencing information exchange in healthcare contexts, and a new theoretical framework to further inform the discipline and science, for use by researchers in future studies. The analytical model and theoretical framework make a significant and new contribution to theory development and understanding of the barriers and facilitators to knowledge exchange in clinical encounters for young people with epilepsy. We hope that researchers will use, refine and develop the model and framework for use in future studies to advance understanding and develop and evaluate new interventions to promote self-care and self-efficacy at transition.

We also identified major deficiencies in current information provision and exchange during the transition period age 13-19 years. The majority of young people reported receiving inadequate information from healthcare professionals and information resources produced by leading epilepsy charities appeared not to be routinely referred to. Consultations with healthcare professionals and young people tended to follow a routine pattern to ascertain information of primary interest to healthcare professionals. Young people felt they could they could not ask questions if they had not followed instructions or done something different, or wanted to know about something not on the usual healthcare professional agenda due to fear of the consequences. Young people wanted to be educated in order to take control of their condition and only one study informed us of how they wished information to be received in order to achieve autonomy [42]. Young people wanted to be seen as a person and not as a disability. They wanted choices, to make decisions and to see healthcare professionals who will listen to them [50].

The expert patient programme (EPP) in the UK, which promotes self-care for adults with long-term conditions [51], has yet to be adapted for children's healthcare, although there are some early pilot programmes funded by the Department of Health. The EPP Community Interactive Company (CIP) commenced 'Staying positive- self management programme' in 2008 for young people with chronic conditions aged between 12-18 years of age [52]. The programme involves workshops aimed to improve their ability to manage their condition be being confident and positive. Evidence from these programme evaluations is urgently anticipated.

There is a complex interplay between several factors that impact on the self-care management practices of young people with epilepsy. Apart from neurological and biological factors, teenagers are a challenging age group to engage with, coupled with the lack of effective interventions and resources to promote information exchange, retention and use. It is clear that service delivery and organisational issues are having a negative impact on the outcomes of young people. Models of service delivery and organisation were not always conducive to promoting effective partnership or information exchange.

According to the Department of Health's Good Practice Guide 2006 [17]*"Transition: moving on well" *young people with neurological impairments receive inadequate services compared to young people with other long-term conditions. Findings from this review indicates that young people disengage with healthcare professionals when age appropriate information is not imparted, lack of direct communication is lacking and feeling of being ignored and lack of continuity is prevalent. This review provides confirmation and further understanding of the barriers and facilitators to information exchange in health care setting and creates a foundation from which to conduct future research.

Finally, we know from our clinical practice that young people with epilepsy can find living with the consequences of epilepsy very challenging. Apart from managing medication, they need to be equipped with life skills to make sometimes unwanted lifestyle adaptations. They also experience similar challenges to other teenagers, such as teenage pregnancy and binge drinking on nights out, but with completely different consequences due to their epilepsy and treatment.

In the spirit of transparency, a number of issues warrant further exploration.

This review focused on the information needs and knowledge exchange of young people with epilepsy at transition. We maintained this focus as the stigma associated with epilepsy, neurological impairments affecting information processing and retention, the specific complexities of epilepsy medication regimes and treatment side effects, and potential consequences of epilepsy in terms of sudden death, required lifestyle modifications, pregnancy and independent living, are unique to epilepsy.

There is a paucity of evidence on effective interventions to promote information exchange, and information retention and use by young people with epilepsy. Nor have available studies followed young people over time to see if interventions showing early promise are effective in the medium to long-term. Young people with epilepsy can find it challenging to remember, understand and use information because of neurological impairments and biological factors. The lack of effective, repetitive and longitudinal interventions and resources for use by this group was a constant feature in the synthesis. Authors also report difficulty in recruiting young people with epilepsy to participate in research studies. More research is urgently required to develop a suite of interventions to support information exchange and use in clinical and community contexts. Where there is some evidence, such as the effectiveness of structured education programmes, further large scale trials are urgently needed.

Parent participants within included studies had children with difficult to control epilepsy and therefore their information needs and experiences may not be typical of parents with children whose epilepsy is reasonably controlled. More mothers than fathers participated in the parent and family studies and there may be key differences in between their information needs and experiences which were not found in this review. There was insufficient evidence to determine whether home background and socio-economic status was an important factor.

We located three studies reporting interventions of teaching young people strategies how to communicate with healthcare professionals and promoting self-care [30-32]. These studies did not identify whether the young people were able to participate in decision making about their epilepsy and future management with healthcare professionals post intervention. Only one study evaluated whether educational programme promoted self-care. Unfortunately the researchers obtained their findings from parent proxy report rather than the young people themselves [30].

## Conclusions

Young people with epilepsy have prolonged and ongoing lifelong contact with health services. Evidence from this review highlights that current models of facilitating information exchange and self-care around transition are not working well and helps explain why healthcare professionals in adult services report that young people with epilepsy enter adulthood ill-equipped and lacking in knowledge or self-care expertise, and sometimes find it difficult to live independently of their parents. Young people are critical of healthcare professional practice, but there are few effective interventions that healthcare professionals can draw on and this urgently needs addressing. Epilepsy is a global problem, but epilepsy research is however critically under-funded and does not receive the same attention as other life-long conditions such as type 1 diabetes.

## Competing interests

The authors declare that they have no competing interests.

## Authors' contributions

SAL and JN were responsible for review questions and design. SAL conducted the electronic and hand search of the literature, SAL and SM conducted the quality appraisal. SAL and JN undertook the analysis, developed the propositions and theoretic framework, and made critical revisions to the paper. JN supervised the study. All authors read and approved the final paper.

## Authors' interests

Sheila Lewis is an Epilepsy Nurse Specialist from the Walton Centre for Neurology and Neurosurgery NHS Foundation Trust.

Professor Jane Noyes is a health services researcher specialising in child health research. She has a specific interest in systematic review methodology and implementation science and is Lead convenor of the Cochrane Qualitative Research Methods Group and Co-Chair of the Cochrane Methods Board Executive, Dr Stephen Mackereth is a Community Paediatrician

## Pre-publication history

The pre-publication history for this paper can be accessed here:

http://www.biomedcentral.com/1471-2431/10/103/prepub
